# Antibacterial activity of royal jelly-mediated green synthesized silver nanoparticles

**DOI:** 10.1186/s13568-021-01213-9

**Published:** 2021-04-01

**Authors:** Susanna Gevorgyan, Robin Schubert, Mkrtich Yeranosyan, Lilit Gabrielyan, Armen Trchounian, Kristina Lorenzen, Karen Trchounian

**Affiliations:** 1 Department of Biochemistry, Microbiology and Biotechnology, Yerevan State University, Alex Manoogian 1, 0025 Yerevan, Armenia; 2 European X-ray Free Electron Laser GmbH, Holzkoppel 4, 22869 Schenefeld, Germany; 3 Institute of Chemical Physics, NAS RA, Paruir Sevak 5/2, 0014 Yerevan, Armenia; 4 Military Aviation University Named After Marshal A. Khamperyants, Arshakunyats 89, 0007 Yerevan, Armenia

**Keywords:** Silver nanoparticles, Green synthesis technology, Royal jelly, Antibacterial activity

## Abstract

The application of green synthesis in nanotechnology is growing day by day. It’s a safe and eco-friendly alternative to conventional methods. The current research aimed to study raw royal jelly’s potential in the green synthesis of silver nanoparticles and their antibacterial activity. Royal jelly served as a reducing and oxidizing agent in the green synthesis technology of colloidal silver nanoparticles. The UV–Vis maximum absorption at ~ 430 nm and fluorescence emission peaks at ~ 487 nm confirmed the presence of Ag NPs. Morphology and structural properties of Ag NPs and the effect of ultrasound studies revealed: (i) the formation of polydispersed and spherical particles with different sizes; (ii) size reduction and homogeneity increase by ultrasound treatment. Antibacterial activity of different concentrations of green synthesized Ag NPs has been assessed on Gram-negative *S. typhimurium* and Gram-positive *S. aureus*, revealing higher sensitivity on Gram-negative bacteria.

## Introduction

Nanoscience is a remarkable field that has the highest potential to improve human life (Ismail et al. 2018; Trchounian et al. 2018). In recent years, nanomaterials' widespread application in various fields of industry, technology, and medicine led to the exponential growth of their global demand (Rónavári et al. 2017; Gabrielyan and Trchounian 2019). Among different metal nanoparticles (NPs), the yearly increase in silver (Ag) NP production was estimated to be hundreds of tons worldwide. The high demand for Ag NPs is conditioned by the commercial utilization of Ag NPs in optics, electronics, catalysis, household items, and a broad range of medical applications (Ge et al. 2014; Gabrielyan and Trchounian 2019). Ag NPs are known for their broad-spectrum antimicrobial and antiviral activities (Gabrielyan and Trchounian 2019; Gabrielyan et al. 2020). These NPs are widely applied in different industries in the disinfection of ailments, water, medical instruments, etc. Also, Ag NPs can play an essential role in preventing epidemics caused by progressive drug-resistant pathogens (Lara et al. 2011).

**Fig. 1:**
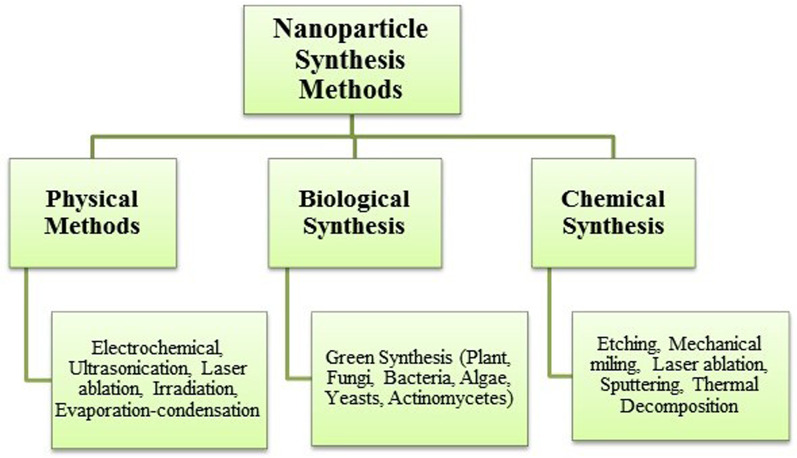
Nanoparticle synthesis methods

Metal NPs synthesis methods are classified as chemical, physical and biochemical synthesis methods (Fig. 1) (Vishwarsrao et al. 2019). Chemical- and physical-based synthesis techniques usually involve toxic organic solvents and hazardous reagents that carry environmental and biological risks and can influence nanoparticle properties, such as toxicity, thus limiting their application in medicine (Forough et al. 2010; Salem et al. 2020). From this point of view, the development of environmentally friendly processes for the synthesis of metal NPs is of great interest.

Green synthesis (GS) strategies of metal NPs are safer alternatives to the conventional chemical and physical methods with a low environmental footprint and mild experimental conditions. It includes mild temperature and pressure and non-toxic, environmentally benign solvents, reducing agents, and capping materials (Kozma et al. 2016; Thunugunta et al. 2015; Narayanan et al. 2011). GS can involve living organisms, such as bacteria (Joerger et al. 2000) or fungi (Moghaddam et al. 2015) and plants or natural extracts containing various biologically active compounds like polysaccharides, proteins, vitamins, or alkaloids. The advantage of listed compounds is that they are generally non-toxic, biodegradable, and can act both as reducing and capping agents, thereby promoting the formation and inhibiting the agglomeration of NPs (Moghaddam et al. 2015; Rónavári et al. 2017).

Venu et al. (2011) described the use of aqueous honey solutions in the synthesis of nanomaterials. From this point of view, the royal jelly (RJ) is an attractive honeybee product. It consists of water (50–60%), proteins (18%), carbohydrates (15%), lipids (3–6%), mineral salts (1.5%), and vitamins (Nagai and Inoue 2004). Also, RJ is composed of many bioactive compounds (Sugiyama et al. 2012). Many studies reported various activities of RJ such as pharmacological activities including antitumor (Swellam et al. 2003), antioxidative (Guo et al. 2007), antimicrobial (Romanelli et al. 2011), vasodilative and hypotensive, anti-fatigue, and anti-allergy, antihypercholesterolemic and anti-inflammatory activities (Fratini et al. 2016). In addition to several physiological effects, RJ is widely used in commercial medical products and food industry (Guo et al. 2007).

RJ's composition is relatively constant at the macro-level. Still, it can vary depending on various factors such as bee nutrition, bee species, harvesting method, the age of the bee larvae, and floral variety, geographical and environmental conditions (Kolayli et al. 2015). From this viewpoint, Armenian honeybee products, including RJ, have unique advantages conditioned by Armenia’s geographical, climatic conditions, and plant diversity.

This study is aimed to evaluate Armenian RJ’s potential as a source of oxidizing and reducing agents in the GS of Ag NPs. In addition to physical–chemical characteristics determined by using UV–Vis and fluorescent spectroscopy, scanning electron microscopy (SEM), transmission electron microscopy (TEM), selected area electron diffraction (SAED), the antibacterial activity of RJ-mediated GS Ag NPs has been assessed on *Salmonella typhimurium* MDC1759 and *Staphylococcus aureus* MDC5233 strains.

## Materials and methods

### Ag NPs synthesis

Raw royal jelly (RRJ) was obtained from the local Armenian beekeeping factory (Province Kotayk, Armenia). As a source of Ag, silver nitrate solution was used. All solutions during the experiment were prepared in doubly distilled water.

For the synthesis of Ag NPs, RRJ aqueous solution in 0.1 g mL^−1^ concentration and 0.5 M silver nitrate solution were used. 1:1 ratio of each solution was mixed under stirring and kept up to 24 h on a magnetic stirrer at room temperature (Mendoza-Reséndez et al. 2014). During the synthesis, the solution’s color started to change from yellowish to brownish color, which indicates the reduction of Ag^+^. The final solution was centrifuged and washed with double distilled water several times. Ag NPs were dispersed in double distilled water using ultrasound (US)—treatment for 20 min (Power of US-homogenizer—50 W; YaXun 2000A).

### Ag NPs characterization

The optical absorption spectrum of Ag NPs was obtained using UV–Vis spectrophotometer (GENESYS 10S UV–Vis, Thermo Scientific, USA) at a resolution of 1 nm between 280 and 720 nm ranges. Fluorescent emission spectra were recorded using a Fluorescence spectrophotometer (Agilent Technologies, USA) at CANDLE Synchrotron Research Institute (Yerevan, Armenia) at different excitation wavelengths.

Morphology, microstructure, and size of GS NPs were observed by scanning electron microscopy (SEM, Leibniz Institute of Photonic Technology, Germany) with 15 kV accelerating voltage, × 15,000–70,000 magnification, secondary electron imaging (SEI) and transmission electron microscopy (JEM-2100-Plus, JEOL, Germany). For TEM measurements, the sample was placed on glow discharged carbon-coated copper grids, incubated, blotted, and dried. Transmission electron micrographs were taken using an accelerating voltage of 200 kV. Selected area electron diffraction (SAED) of the NPs was also recorded using the TEM. TEM experiments were conducted in the XBI Biolab of European XFEL (Han et al. 2021). Average Ag core diameter, size distributions were calculated for each sample by averaging ~ 260 NPs from the TEM images using ImageJ software.

The influence of US-treatment on the sizes and distribution of NPs was studied by using Atomic Force Microscopy (AFM, Solver Nano NT-MDT ISN, Laboratory of “Heliotechnics”, National Polytechnic University of Armenia, Yerevan).

### Assessment of antibacterial activity

The study of antibacterial activity of GS and ultrasound-treated Ag NPs was performed on *S. typhimurium* MDC1759 and *S. aureus* MDC5233 strains (Microbial Depository Center, National Academy of Science, Yerevan, Armenia). The mentioned strains were grown in Nutrient Broth (NB) media at 37 °C and pH 7.5 (Gabrielyan et al. 2020). Anaerobic conditions were maintained as described (Gabrielyan et al. 2020). These strains were cultivated in the presence of GS and ultra-sonicated Ag NPs (from 5 to 20 μL mL^−1^ v/v ratio). The growth of bacteria was measured by densitometer (DEN-1B McFarland, Biosan, Latvia) for 6 h of growth. The specific growth rate of bacteria was calculated using the following formula: growth rate = (lnOD_t_ – lnOD_0_)/t, where OD_0_ (optical density) is the initial value of OD_600_; OD_t_ is the value of OD_600_ after t hours (Gabrielyan et al. 2020).

### Statistical analysis

Experiments were repeated three times. The results are represented as means ± SD. Standard errors, as well as the validity of the differences between different series of experiments, were evaluated by Student’s validity criteria (*P*), were calculated by using the appropriate functions of Microsoft Excel 2010.

## Results

### UV–Vis spectroscopy analysis

**Fig. 2:**
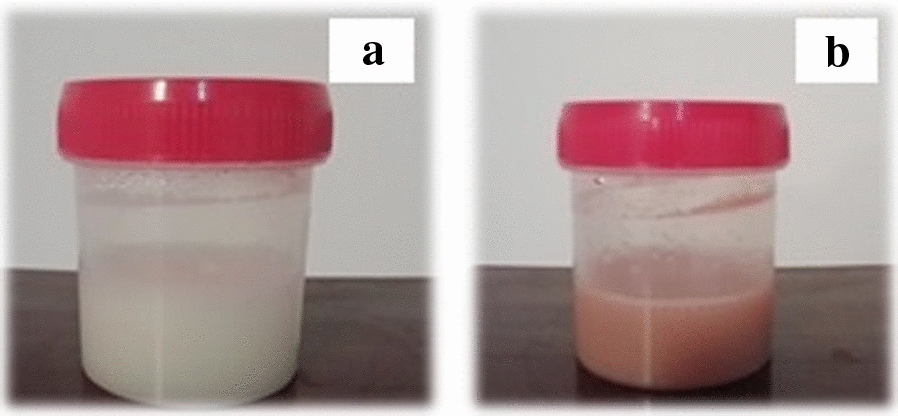
Initial mixed solutions of RJ and AgNO_3_ before (**a**) and after incubation (**b**)

**Fig. 3:**
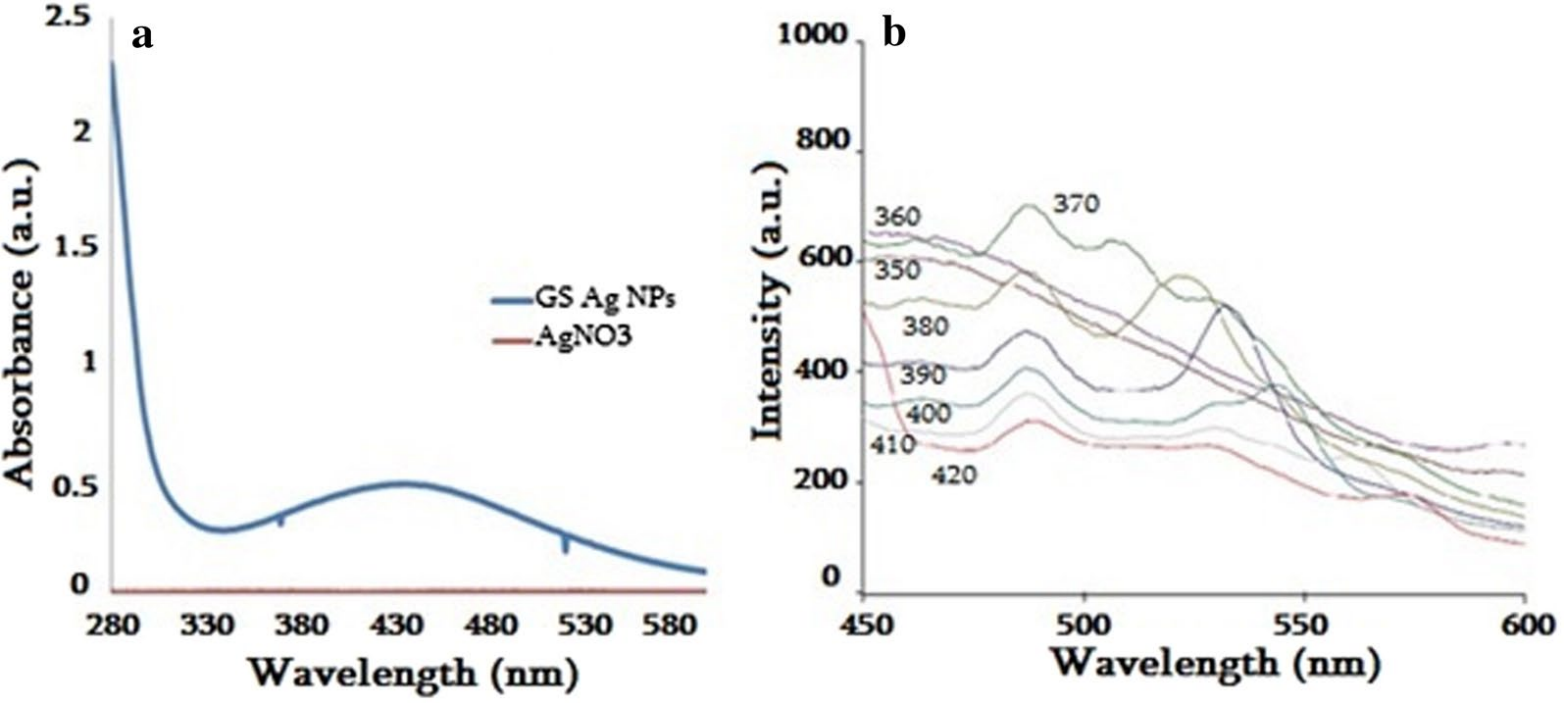
UV–Vis absorption spectra (**a**) and fluorescence emission spectra (**b**) of GS Ag NPs

Change in color of the RJ solution mixed with AgNO_3_ solution from yellowish to brownish color visually observed, indicating Ag ions’ reduction (Fig. 2a, b). After 24 h stirring final solution was centrifuged and washed with distilled water several times. Absorption measurements by UV–Vis spectrophotometer revealed the maximum absorption at ~ 430 nm, confirming the presence of Ag NPs (Fig. 3a).

### Fluorescence spectroscopy

The fluorescence emission spectra of GS Ag nanostructures were obtained using from 370 to 420 nm excitation wavelengths, which are indicated on corresponding curves (Fig. 3b). Emission peak at ~ 487 nm confirmed the presence of Ag nanostructures. By the increase of excitation wavelength, redshifted additional emission peaks were observed, which may be conditioned by the polydispersity of the NPs sample.

### Electron microscopy

**Fig. 4:**
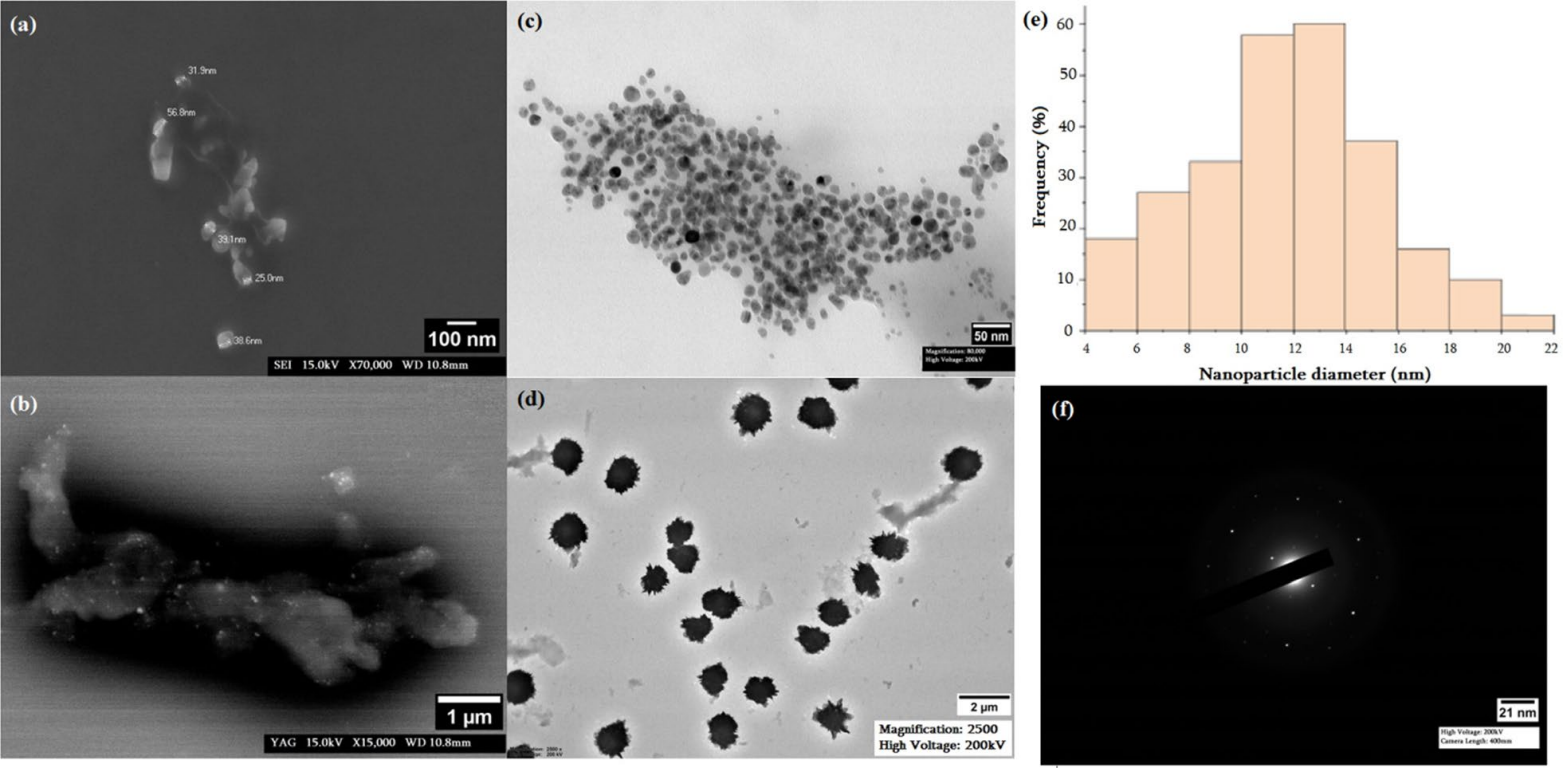
SEM (**a**, **b**), TEM (**c**, **d**) micrographs and nanoparticle diameter distribution (**e**) and SAED pattern (**f**)

SEM and TEM were used to examine the morphology and microstructure of synthesized particles. Scanning electron micrograph revealed more or less spherical morphology of GS Ag NPs and showed that particles are coated by polymer (bright dots on SEM micrographs; Fig. 4a, b).

The analysis of data from TEM micrographs provided further insight into the morphology and particle size. It confirmed the formation of polydispersed and spherical nanostructures of different sizes (Fig. 4c, d). Two different sample species could be identified. A small size species of GS Ag NPs with a particle size distribution are shown in the histogram in Fig. 4e. The average diameter of the spherical NPs was 11.7 ± 3.58 nm. And a second species containing a few large clusters up to 1 μm was observed (see Fig. 4d), which can be a result of the aggregation of small nanostructures.

### Atomic force microscopy

**Fig. 5:**
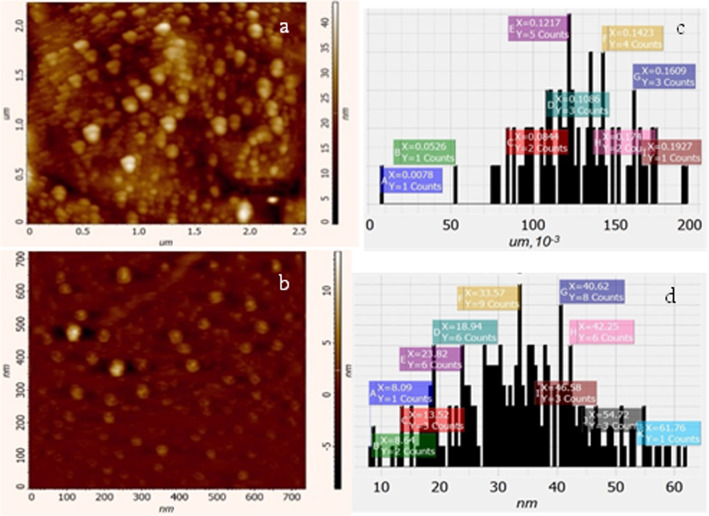
AFM images of non-treated (ultrasound-treated **a** (**b**)) and average size distribution **c** (**d**) of Ag NPs

The effect of ultrasound treatment on GS Ag NPs was analyzed by AFM, and obtained data revealed that ultrasonication contributes to the homogenization of Ag NPs’ colloidal solution and reduces the size of nanostructures (Fig. 5). For the purposes of comparative analysis, AFM images of Ag NPs without and with ultrasound-treatment as well as the size of the corresponding nanoparticles distributions are shown (see Fig. 5), representing 2D topography of the NPs without and with US treatment, respectively. The data shows the distributions of particle sizes both without and with US treatment cases. Statistical analysis shows that before and after US-treatment NPs have sizes of 7.8–192.7 nm and 8.6–61.8 nm, respectively. Furthermore, over 90% of the NPs have sizes in the interval of 52–174.0 nm and 8.9–54.7 nm, respectively. The average values of some topographical parameters are presented in Table 1*.* As a result, it can be said that US-treatment leads to a more homogeneous distribution and a significant reduction in the size of NPs.

**Table 1:** Average topographical (error values: ± 5%) parameters of GS Ag NPs with and without US-treatment

Ag NPs	Size, Nm	Length, Nm	Height, nm	Area nm*nm	Volume nm*nm*nm
Without US-treatment	126.010	191.051	24.12	16,801.001	129 × 10^6^
With US-treatment	34.003	53.068	13.03	1279.048	3385.355

### Antibacterial activity

**Fig. 6:**
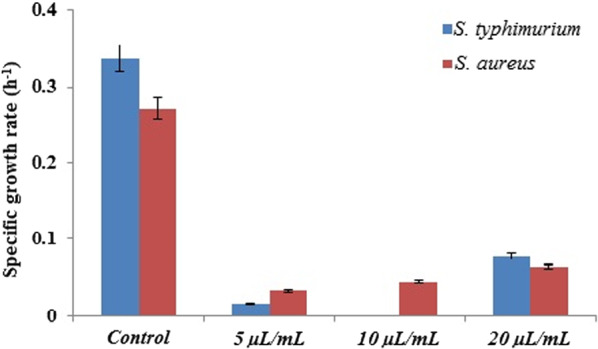
Specific growth rate of *S. typhimurium* MDC1759 and *S. aureus* MDC5233, cultivated without (control) and with addition of different concentrations of GS Ag NPs (p < 0.05)

**Fig. 7:**
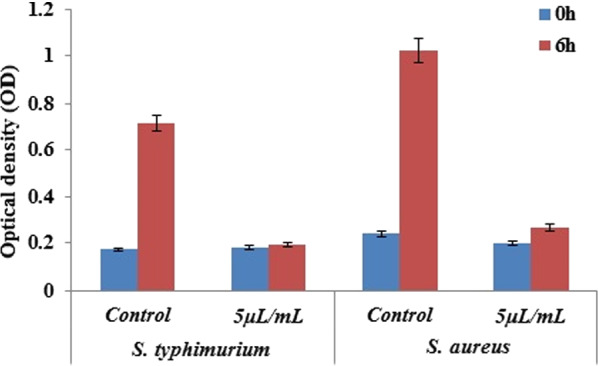
Changes of optical density of bacteria, grown without (control) and with addition of GS Ag NPs (p < 0.05)

The growth properties of *S. typhimurium* MDC1759 and *S. aureus* MDC5233 strains, cultivated under anaerobic conditions, in the presence of GS and ultra-sonicated Ag NPs (from 5 to 20 μL per mL) have been investigated. The control was bacteria grown without NPs addition. The bactericidal effect for both bacteria was observed at all concentrations of NPs (Figs. 6, 7). Moreover, RJ-mediated Ag NPs display more expressed antimicrobial effect at low concentration (Fig. 6). Similar result was obtained with *S. typhimurium* MDC1759 in the presence of commercial Ag NPs (Gabrielyan et al. 2020).

The growth data analysis showed that Gram-negative *S. typhimurium* had higher sensitivity than Gram-positive *S.* aureus, which may be conditioned by the membrane structure—a narrower cellular wall of these bacteria (Aghajanyan et al. 2020; Meikle et al. 2020). The antibacterial activity of RJ-mediated GS Ag NPs can be a result of interaction of NPs with bacterial membranes and their penetration into the bacterial cell (Trchounian et al. 2018; Gabrielyan et al. 2020).

## Discussion

It is well known that Ag ions and Ag-based compounds, including Ag NPs, exhibit a toxic effect on both Gram-negative and Gram-positive microorganisms (Losasso et al. 2014). The exact mechanism of antibacterial activity of Ag NPs has not been entirely clarified, but various hypotheses have been proposed. The action of Ag NPs can be conditioned by free Ag ions, present or released from the nanomaterials, which can bind to the cell membrane, destabilize membrane potential and lead to disruption of the bacterial envelope (Bapat et al. 2018; Yin et al. 2020). Free silver ions also can induce the generation of reactive oxygen species as well as inhibit the synthesis of proteins by denaturing ribosomes (Durán et al. 2016). Different capping agents, including biologically active compounds as well as the presence of organic and inorganic components in media, can change the dissolution behavior of NPs, thus influencing the release of silver ions and antibacterial activity strength of Ag NPs (Yin et al. 2020). According to Konovalov et al. (2013), biologically active compounds (BACs) as a capping agent can have a great impact on efficient concentration. BACs, especially in the case of GS of Ag NPs, can lead to the formation of nanoassociates in highly diluted samples, which will increase the efficiency. This may also be conditioned by the higher diffusion rate of low concentrated solutions compared to highly concentrated solutions, which can form aggregates (Konovalov et al. 2013). On the other hand, antimicrobial activities of many peptides present in RJ were demonstrated (Romanelli et al. 2011; Li et al. 2012). The action mechanism of these antimicrobial peptides is due to the change of cell membrane permeability; particularly they cause decrease of lipid layer surface and lead to the membrane disruption or create pores thus destabilizing membrane (Fratini et al. 2016; Li et al. 2012). Cell wall structure also plays a crucial role in the antibacterial activity of Ag NPs. Gram-negative bacteria are more susceptible to Ag NPs due to narrower cellular walls compared to Gram-positive strains (Aghajanyan et al. 2020; Meikle et al. 2020).

TEM revealed the presence of small as well as clustered NPs. US-treatment leads to a decrease in the size and an increase in homogeneity of the distribution of GS NPs and contributes to the higher antibacterial activity against Gram-negative *S. typhimurium* and Gram-positive *S. aureus*, in that Gram-negative was more susceptible*.* The results suggest that green synthesis plays an important role in optimizing properties and biological activity as well as the inhibitory efficiency of synthesized Ag NPs, while to better understand the antibacterial activity mechanism of GS Ag NPs, further research is needed.

## Data Availability

All data generated or analysed during this study are included in this article.

## Abbreviations

GS: Green synthesis
RJ: Royal jelly
Ag NPs: Silver nanoparticles
SEM: Scanning electron microscopy
TEM: Transmission electron microscopy
AFM: Atomic force microscopy
US: Ultrasonication
SAED: Selected area electron diffraction
OD: Optical density
MDC: Microbial depository center
